# Adipose mesenchymal stem cells from osteoporotic donors preserve functionality and modulate systemic inflammatory microenvironment in osteoporotic cytotherapy

**DOI:** 10.1038/s41598-018-23098-8

**Published:** 2018-03-26

**Authors:** Chen-Xi Zheng, Bing-Dong Sui, Nu Liu, Cheng-Hu Hu, Tao He, Xin-Yi Zhang, Pan Zhao, Ji Chen, Kun Xuan, Yan Jin

**Affiliations:** 1 State Key Laboratory of Military Stomatology & National Clinical Research Center for Oral Diseases & Shaanxi International Joint Research Center for Oral Diseases, Center for Tissue Engineering, School of Stomatology, Fourth Military Medical University, Xi’an, Shaanxi 710032 China; 2 Research and Development Center for Tissue Engineering, Fourth Military Medical University, Xi’an, Shaanxi 710032 China; 3 Xi’an Institute of Tissue Engineering and Regenerative Medicine, Xi’an, Shaanxi 710032 China

## Abstract

Maintenance of bone homeostasis against diseased microenvironments remains as a major challenge. Recently, mesenchymal stem cells (MSCs) have been unravelled as potent microenvironmental modulators, the systemic infusion of which in cytotherapy can prevent or rescue extensive bone loss via anti-inflammation. However, MSCs also accept microenvironmental regulations; particularly, MSCs from bone marrow (BMMSCs) are prone to pathological microenvironmental factors of bone. In this study, we discovered that BMMSCs from osteoporotic donors of ovariectomized (OVX) mice lost their anti-inflammatory capability and failed to prevent bone loss when infused back into OVX recipients. Nevertheless, MSCs from adipose tissues (ADMSCs) preserved their anti-inflammatory capacity, despite diseased microenvironments of OVX donors, and continued to show protective effects on bone in OVX recipients. In the cellular level, the anti-inflammatory superiority of osteoporotic donor-derived ADMSCs over BMMSCs existed in their distinctive capability to induce T-cell apoptosis, which was molecularly attributed to retained expression levels of critical immunomodulatory genes. Furthermore, these functional discrepancies of BMMSCs and ADMSCs were due to differential stemness, energy metabolism and anti-oxidative defence system, underlying general disparity in their cellular states. Collectively, our findings optimize osteoporotic cytotherapy by using ADMSCs in resistance to and in modulation of diseased microenvironments.

## Introduction

Maintenance of postnatal bone homeostasis requires dynamic bone remodelling balance carefully controlled by local and circulatory microenvironments^1,2^. In pathological conditions, microenvironmental alterations such as estrogen deficiency and the associated inflammation trigger extensive bone loss, the cure to which remains as an unfulfilled challenge in modern medicine^3–5^. In the recent decade, other than their putative role in keeping tissue homeostasis, mesenchymal stem cells (MSCs) have emerged as potent microenvironmental modulators, the systemic infusion of which exerts immense anti-inflammatory effects that benefit a variety of tissues/organs including bone^6,7^. Indeed, we and others have revealed the efficacy of systemic MSC therapy to restore bone remodelling in prevention or treatment of osteoporosis, via normalizing the diseased inflammatory microenvironments rather than exerting local effects by homing to osteoporotic region^8,9^. However, as reciprocal interactions, MSCs also accept microenvironmental regulations; particularly, MSCs from bone marrow (BMMSCs) are prone to pathological factors of bone, demonstrating impaired function including unstable anti-inflammatory efficacy in recipient bone loss, which hinders their therapeutic applications^2,9^. Therefore, optimizing MSC therapy by establishing novel strategies to resist and guarantee modulation against diseased microenvironments is of great significance for improved approaches to osteoporosis.

Intriguingly, it has been documented that MSCs from diverse origins exhibit functional preferences and differences in health and diseases^10–12^. In particular, MSCs from adipose tissues (ADMSCs) demonstrate functional maintenance in certain conditions, potentially underlying increased adiposity observed in aged and postmenopausal osteoporotic individuals^13,14^. Indeed, functional discrepancies of BMMSCs and ADMSCs from estrogen-deficient and aged osteoporotic donors have been revealed *in vitro*, in that ADMSCs, but not BMMSCs, preserve cell viability and differentiation capacities^3,12,15–20^. In addition to these *in vitro* findings, less affected regenerative potential of ADMSCs was also confirmed using local transplantation in aged and OVX bone loss and defects^21–23^. Nevertheless, the above studies only focused on behavioural outcomes of MSCs themselves. Whether osteoporotic donor-derived BMMSCs or ADMSCs resist and further modulate diseased microenvironments in systemic cytotherapy are still unknown. Given that topical administration of MSCs directly into bone marrow will cause invasive injuries^21,23^, and that anti-inflammation rather than homing contributes to therapeutic efficacy of systemically delivered MSCs especially in OVX-induced osteoporosis^8,9^, further elucidating performances and mechanisms of BMMSCs and ADMSCs in resistance to and in modulation of diseased microenvironments in this model would provide valuable information and solutions to optimize osteoporotic cytotherapy.

In this study, according to the above stated intention, we discovered that BMMSCs from OVX osteoporotic donors lost their anti-inflammatory capability and failed to prevent bone loss when infused back into OVX recipients. Nevertheless, as a promising alternative, ADMSCs preserved their anti-inflammatory capacity, despite diseased microenvironments of OVX donors, and continued to show protective effects on bone mass and bone remodelling balance in OVX recipients upon systemic delivery. Mechanistically, the anti-inflammatory superiority of osteoporotic donor-derived ADMSCs over BMMSCs existed in their distinctive capability to induce T-cell apoptosis, which was attributed to retained expression levels of critical immunomodulatory genes and was further due to maintained stemness, energy metabolism and anti-oxidative defence system. Collectively, these results indicated that ADMSCs with general maintenance of cellular states can resist to diseased microenvironments and act as an optimal source for osteoporotic cytotherapy via exerting microenvironmental modulatory effects.

## Results

### ADMSCs from osteoporotic donors preserve efficacy to prevent estrogen deficiency-induced osteoporosis

**Figure 1:**
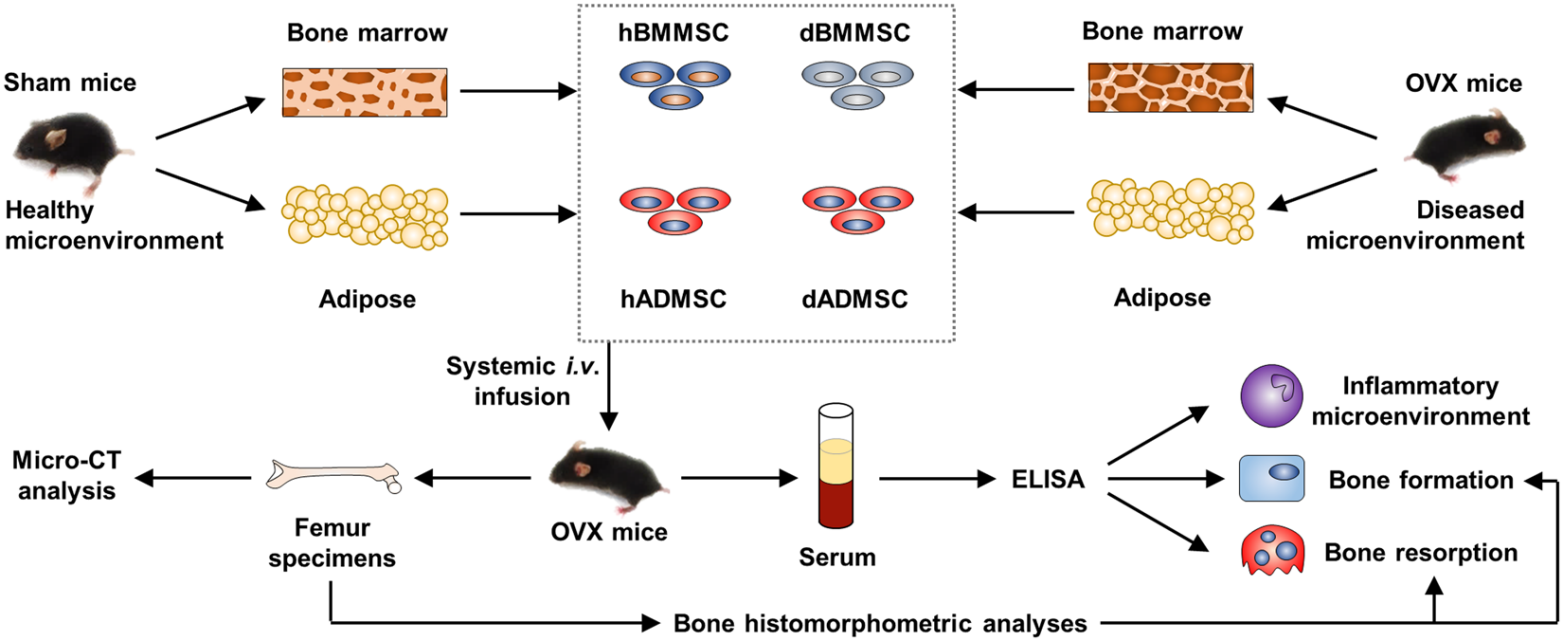
Schema indicating the study design for MSC isolation, transplantation and outcome evaluation. MSCs were isolated after 4 weeks of donor Sham or OVX surgeries, and were infused at 7 days post to recipient OVX operation. Recipient mice were sacrificed at 21 days after infusion.

To explore potential functional discrepancies of MSCs from different origins in response to and in modulation of diseased microenvironments in osteoporotic cytotherapy particularly with donor comorbidities, we isolated BMMSCs and ADMSCs from both Sham and OVX-induced osteoporotic mice (Fig. 1). As reported, the OVX model represents the skeletal pathogenesis triggered by microenvironmental alterations of estrogen deficiency and the secondary inflammation^3,24^. Accordingly, we intravenously infused BMMSCs and ADMSCs from OVX-induced diseased donors (*i*.*e*. dBMMSCs and dADMSCs) and their respective healthy counterparts (*i*.*e*. hBMMSCs and hADMSCs) into OVX-induced diseased recipients (Fig. 1). The MSCs we used were in consistent with the current MSC standard for therapeutic applications (Supplementary Figure S1)^2,9,25^.

**Figure 2:**
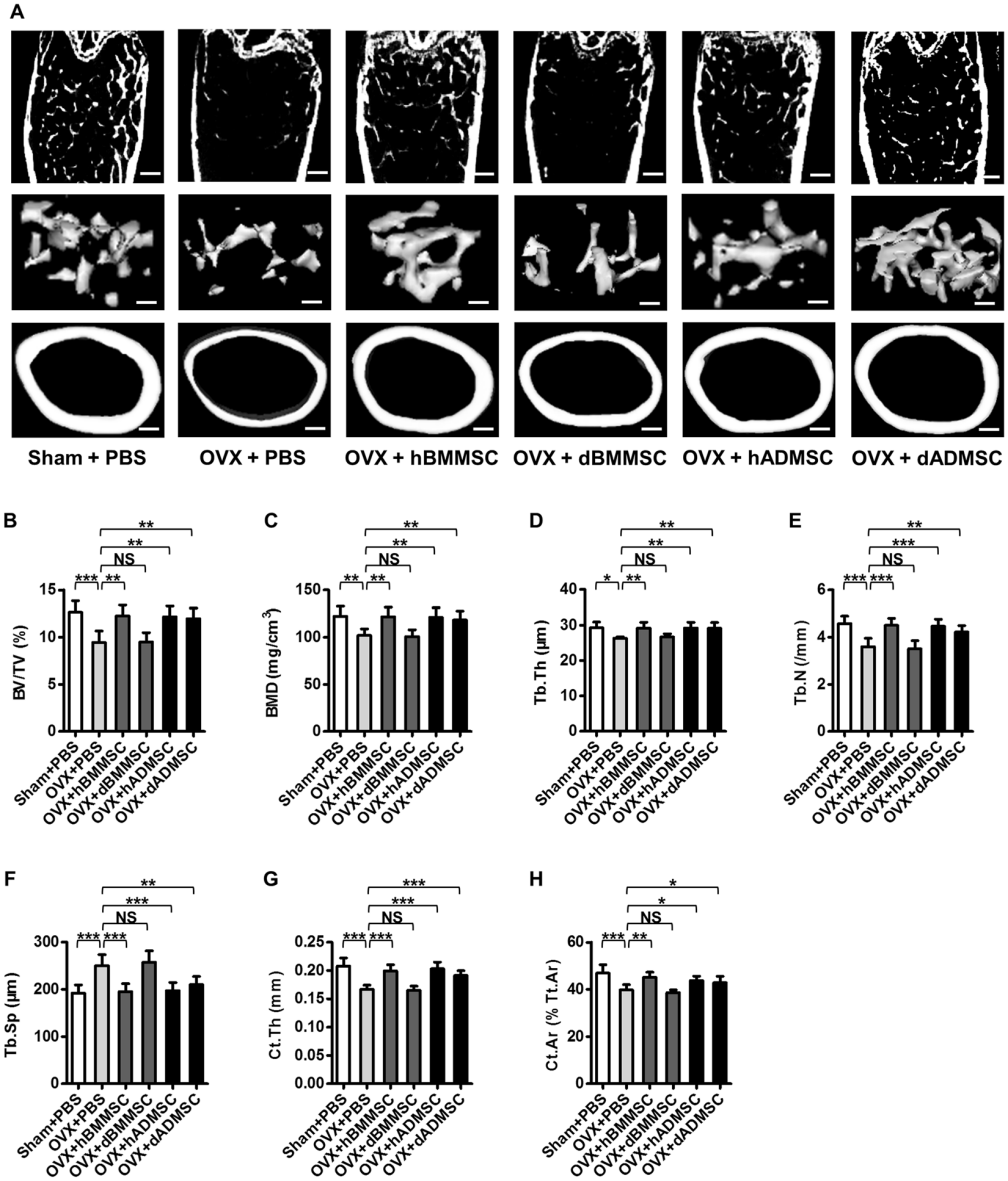
Effects of systemic infusion of Sham and osteoporotic donor-derived MSCs on recipient osteoporosis. (**A**) Representative micro-CT images of total (top), trabecular (middle) and cortical (bottom) bone mass of femora harvested at D28 post operation. Bars: 400 μm (top and bottom) and 150 μm (middle). (**B–H**) Quantitative analysis of trabecular (**B–F**) and cortical (**G**,**H**) bone microarchitecture in the distal metaphyses of femora. *n* = 6 per group. Data are shown as mean ± SD. *P < 0.05, **P < 0.01 and ***P < 0.001. NS, not significant (*P* > 0.05). Data were analysed using ANOVA followed by Newman-Keuls post-hoc tests.

For the translational significance, efficacy of the infused MSCs to prevent bone loss was firstly examined by micro-computed tomography (micro-CT) analysis at 28 d post OVX operation (Fig. 1), according to previous studies^8,25^. Data demonstrated that systemic transplantation of hBMMSCs and hADMSCs both effectively prevented trabecular and cortical bone loss and bone microarchitecture impairments, as indicated by representative micro-CT images (Fig. 2A) and corresponding trabecular (Fig. 2B–F) and cortical (Fig. 2G,H) bone parameters. Furthermore, effects of hBMMSCs and hADMSCs were comparable. However, BMMSCs derived from osteoporotic donors failed to protect bone mass, but dADMSCs continued to prevent OVX-induced bone loss, demonstrating paralleled efficacy to hBMMSCs and hADMSCs (Fig. 2). These findings indicated that ADMSCs from osteoporotic donors resisted to diseased host microenvironments and preserved efficacy to prevent estrogen deficiency-induced osteoporosis, while dBMMSCs lost their efficacy in cytotherapy.

### ADMSCs from osteoporotic donors maintain efficacy to hold bone remodelling balance

**Figure 3:**
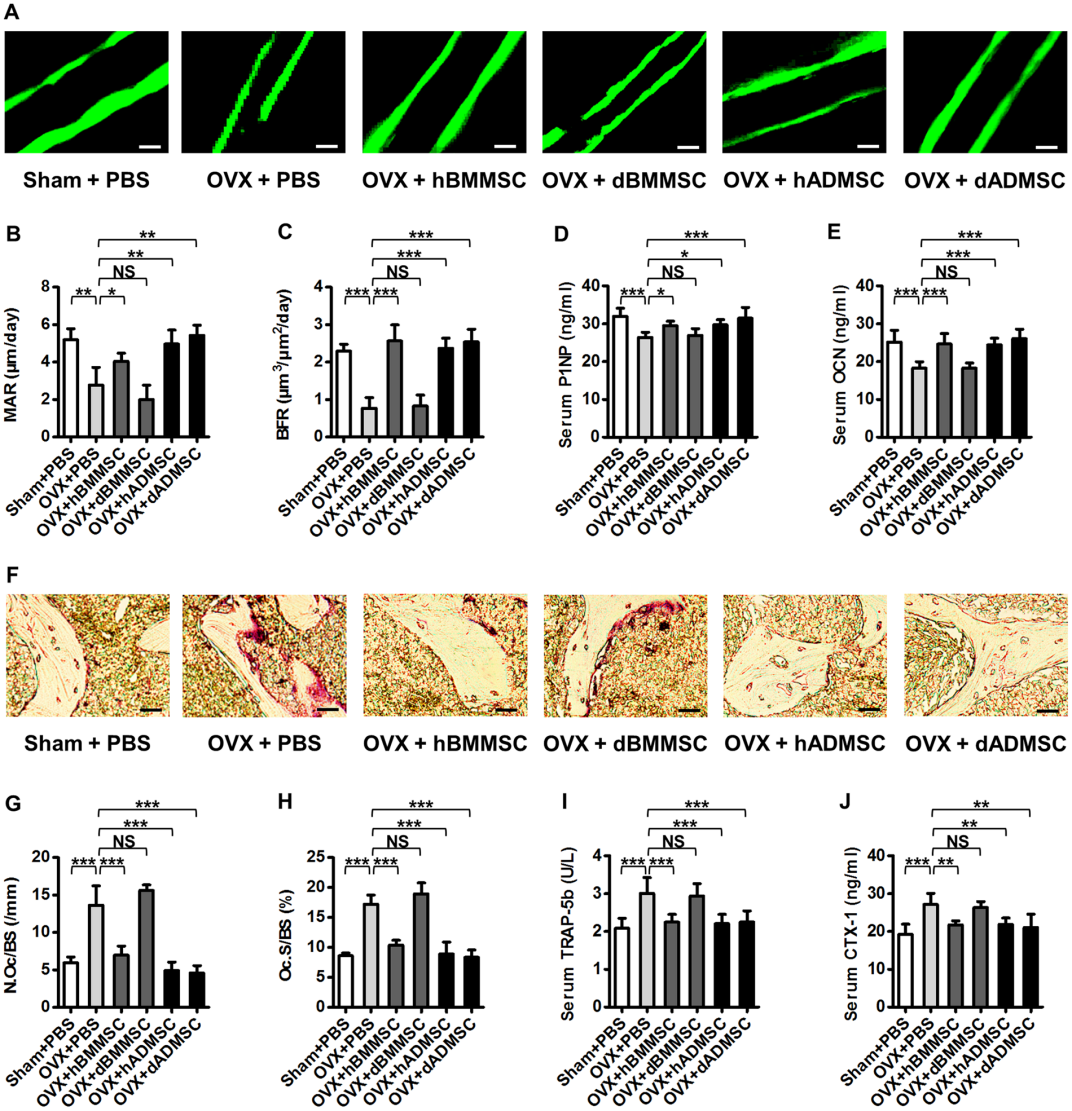
Bone remodelling changes after infusion of Sham and osteoporotic donor-derived MSCs. (**A–C**) Representative images of calcein labelling (**A**) and parameters of bone formation rates (**B**,**C**) in distal femora harvested at D28 post operation. Bars: 50 μm. *n* = 3 per group. (**D**,**E**) ELISA analysis of serum levels of bone formation markers P1NP (**D**) and OCN (**E**). *n* = 6 per group. (**F–H**) Representative images of TRAP staining (**F**) and parameters of bone resorption rates (**G**,**H**) in distal femora harvested at D28 post operation. Bars: 25 μm. *n* = 3 per group. (**I**,**J**) ELISA analysis of serum levels of bone resorption markers TRAP-5b (**I**) and CTX-1 (**J**). *n* = 6 per group. Data are shown as mean ± SD. *P < 0.05, **P < 0.01 and ***P < 0.001. NS, not significant (*P* > 0.05). Data were analysed using ANOVA followed by Newman-Keuls post-hoc tests.

It has been revealed that osteoporotic bone loss is generally attributed to the imbalance between bone formation and bone resorption rates^1^, which could be rescued by systemic transplantation of healthy donor-derived ADMSCs and BMMSCs^9^, as also shown by our data of bone histomorphometric and serological analyses (Fig. 3). Therefore, we next investigated whether BMMSCs and ADMSCs derived from osteoporotic donors possessed functional discrepancies in intervening bone remodelling balance (Fig. 1). Double calcein labelling (Fig. 3A) and the corresponding parameters (Fig. 3B,C) demonstrated that dADMSCs, but not dBMMSCs, maintained bone formation rates in OVX mice, consistent with serological analysis of bone formation markers Procollagen 1 N-terminal peptide (P1NP) (Fig. 3D) and Osteocalcin (OCN) (Fig. 3E). Besides, tartrate-resistant acid phosphatase (TRAP) staining (Fig. 3F) as well as related parameters (Fig. 3G,H) further revealed that dADMSCs, but not dBMMSCs, suppressed bone resorption activation in OVX mice, which was further confirmed by serological analysis of bone resorption markers Tartrate-resistant acid phosphatase iso-form 5b (TRAP-5b) (Fig. 3I) and Cross linked C-telopeptide of type 1 collagen (CTX-1) (Fig. 3J). In summary, histomorphometric and serological data demonstrated that dADMSCs indeed exerted both anabolic and anti-catabolic effects on recipient osteoporosis, while dBMMSCs were not effective, despite comparable efficacy of hBMMSCs and hADMSCs (Fig. 3). These results suggested that ADMSCs from osteoporotic donors maintained efficacy to hold bone remodelling balance against recipient osteoporosis.

### Osteoporotic donor-derived ADMSCs keep anti-inflammatory capability to modulate diseased recipient microenvironments

**Figure 4:**
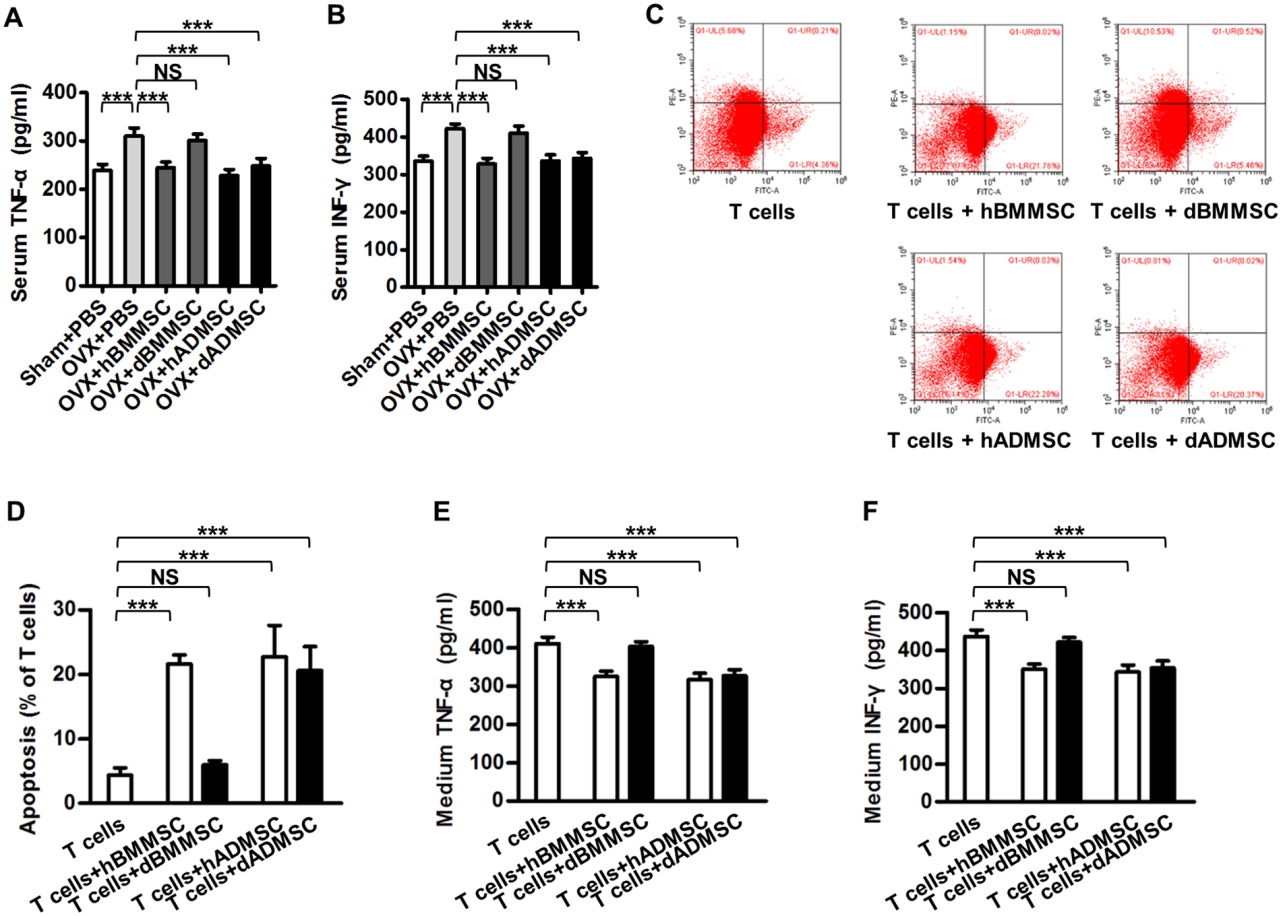
Anti-inflammatory capability of Sham and osteoporotic donor-derived MSCs. (**A**,**B**) ELISA analysis of serum levels of inflammation markers TNF-α (**A**) and IFN-γ (**B**). (**C**,**D**) Representative images (**C**) and quantitative analysis (**D**) of T-cell apoptosis induced by MSCs. MSCs were used at the 1^st^ passage, and stimulated T cells were either cultured without MSCs or directly added onto MSCs for 6 h. The apoptotic rate of T cells was calculated by percentages of early apoptotic (FITC^+^PI^-^) plus late apoptotic/necrotic (FITC^+^PI^+^) cells. (**E**,**F**) ELISA analysis of TNF-α (**E**) and IFN-γ (**F**) in media from T cells cultured or co-cultured with MSCs. *n* = 6 per group. Data are shown as mean ± SD. ***P < 0.001. NS, not significant (*P* > 0.05). Data were analysed using ANOVA followed by Newman-Keuls post-hoc tests.

MSCs prevent or alleviate osteoporosis through systemic effects that modulate microenvironment^9^ or local effects such as differentiation into osteoblasts^25^. Given that only a few of both systemically infused ADMSCs and BMMSCs homed to OVX recipient bone marrow within 24 h and 3 d after transplantation (Supplementary Figure S2) and that previous study has demonstrated that most MSCs could not be detected a few weeks later^9^, we proposed that MSCs rescued bone remodelling in estrogen deficiency-induced osteoporosis mainly through modulation of recipient systemic microenvironments. We and others have documented that Tumor necrosis factor-alpha (TNF-α) and Interferon-gamma (IFN-γ) are the key inflammatory cytokines impairing bone and MSCs^3,26^, and that anti-inflammation of MSCs is a critical mechanism underlying their therapeutic effects on osteoporosis^8,9^. Specifically, MSCs exert anti-inflammation effects mainly through decreasing T cells numbers via induction of apoptosis^7–9^. As to the comparison between BMMSCs and ADMSCs, we first found that healthy donor-derived BMMSCs and ADMSCs comparably reduced CD3^+^ T cells in peripheral blood of osteoporotic mice (Supplementary Figure S3) and decreased inflammation cytokines levels (Fig. 4A,B), indicating comparable anti-inflammation abilities between BMMSCs and ADMSCs. Next, we deciphered whether BMMSCs and ADMSCs derived from diseased donor microenvironments could resist and modulate diseased recipient systemic microenvironments in osteoporotic cytotherapy. We analysed serological levels of inflammatory cytokines after infusion of MSCs into OVX mice (Fig. 1), and discovered that dADMSCs, together with hBMMSCs and hADMSCs, significantly suppressed TNF-α (Fig. 4A) and IFN-γ (Fig. 4B) levels in recipient serum, but dBMMSCs did not show anti-inflammatory effects. We further investigated and found that except dBMMSCs, dADMSCs with hBMMSCs and hADMSCs substantially induced apoptosis of stimulated T cells (Fig. 4C,D), with associated decrease of TNF-α and IFN-γ secretion levels in conditional media, confirming the *in vivo* findings (Fig. 4E,F).

**Figure 5:**
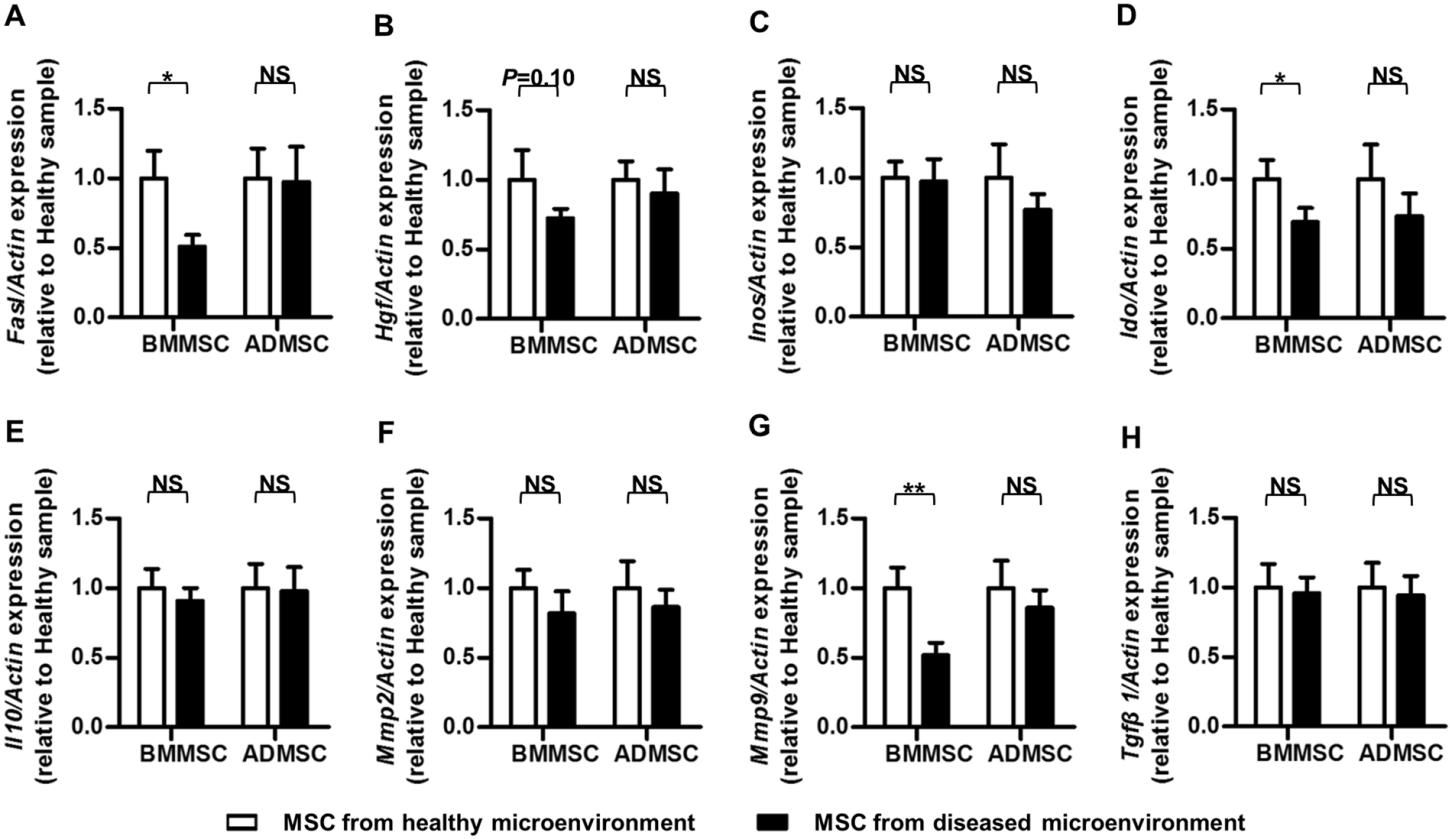
Immunomodulatory properties of MSCs from healthy (Sham donors) and diseased (OVX donors) microenvironments. MSCs were used at the 1^st^ passage, and stimulated T cells were directly added onto MSCs for 6 h. MSC samples were then gathered for qRT-PCR analysis of the mRNA expression levels of immunomodulation marker genes *Fasl* (**A**), *Hgf* (**B**), *Inos* (**C**), *Ido* (**D**), *Il10* (**E**), *Mmp2* (**F**), *Mmp9* (**G**) and *Tgfβ1* (**H**). The relative mRNA expression levels of osteoporotic donor-derived MSCs were obtained by normalizing first against *β-actin* and then against those of the respective healthy donor-derived MSCs. *n* = 6 per group. Data are shown as mean ± SD. *P < 0.05 and **P < 0.01. NS, not significant (*P* > 0.05). Data were analysed using two-tailed Student’s t-test for BMMSCs or ADMSCs, respectively.

Molecularly, to understand potential meditators underlying the anti-inflammatory changes, we further examined the mRNA expression profile of the reported immunomodulatory factors in MSCs^9^ (Fig. 5). Quantitative real-time polymerase chain reaction (qRT-PCR) results demonstrated that the expression levels of *Fas ligand* (*Fasl*)^7,8^, *Indoleamine 2*,*3-dioxigenase* (*Ido*)^27^ and *Matrix metalloproteinase 9* (*Mmp9*)^28^ were maintained in osteoporotic donor-derived ADMSCs while down-regulated in BMMSCs of the same donors. Besides, the expression level of *Hepatocyte growth factor* (*Hgf*)^29^ showed similar tendency, though without statistical significance. In addition, no significant difference was detected regarding the expression levels of *Interleukin-10* (*Il10*)^30^, *Inducible nitric oxide synthase* (*Inos*)^6^, *Matrix metalloproteinase 2* (*Mmp2*)^28^ and *Transforming growth factor-beta 1* (*Tgfβ1*)^31^ between MSCs derived from Sham and osteoporotic donors (Fig. 5). Together, these results suggested that compared to BMMSCs, anti-inflammatory capability of ADMSCs was rarely affected by diseased donor microenvironments, which reciprocally contributed to the preserved modulatory effects on diseased recipient microenvironments in osteoporotic cytotherapy.

### Retained stemness, metabolism and anti-oxidants of ADMSCs underlie preserved function in diseased microenvironments

To further identify preserved function of ADMSCs derived from diseased donor microenvironments, we evaluated major behavioural aspects of MSCs in cell viability and differentiation. As depicted, estrogen-deficient microenvironments and the related systemic inflammation significantly reduced colony-forming capability and proliferation rates of BMMSCs, but not ADMSCs, with corresponding changes in mRNA expression levels of proliferation-related genes *Cyclin D1 (Ccnd1)*, *Cyclin D2 (Ccnd2)*, *Cyclin E1 (Ccne1)* and senescent marker gene *P53*^32^ (Supplementary Figure S4). Moreover, both osteogenic and adipogenic potential of ADMSCs was maintained despite osteoporotic donors, while dBMMSCs exhibited the differentiation switch from osteogenesis to adipogenesis, as confirmed by qRT-PCR analysis on osteogenic markers *Alkaline phosphatase* (*Alp*) and *Runt-related transcription factor 2* (*Runx2*) and adipogenic markers *Peroxisome proliferator activated receptor gamma* (*Pparγ*) and *CCAAT/enhancer binding protein alpha* (*C/ebpα*) (Supplementary Figure S5). These findings collectively indicated functional resistance of ADMSCs to diseased donor microenvironments in osteoporosis.

**Figure 6:**
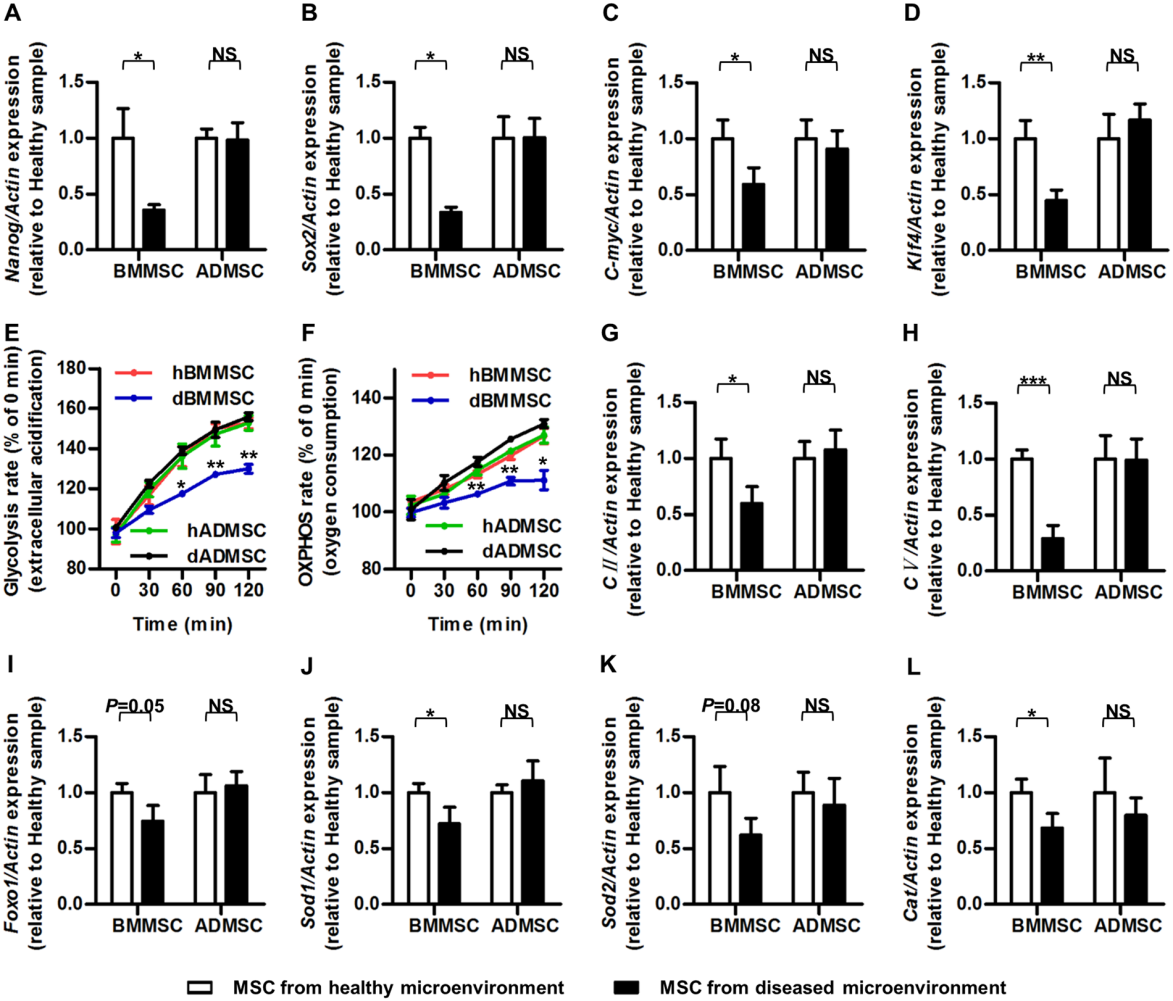
Stemness and metabolism of MSCs from healthy (Sham donors) and diseased (OVX donors) microenvironments. (**A–D**) qRT-PCR analysis of the mRNA expression levels of stemness genes *Nanog* (**A**), *Sox2* (**B**), *C-myc* (**C**) and *Klf4* (**D**). (**E**) Glycolysis rate of MSCs. Glycolysis rate was determined via assessment of extracellular acidification by a fluorescence probe. Fluorescence values of each timepoint were normalized to those of 0 min. (**F**) OXPHOS rate of BMMSCs and ADMSCs. OXPHOS rate was determined via assessment of extracellular O_2_ by a fluorescence probe. Fluorescence values of each timepoint were normalized to those of 0 min. (**G**,**H**) qRT-PCR analysis of the mRNA expression levels of mitochondrial energy metabolism genes *CII* (**G**) and *CV* (**H**). (**I–L**) qRT-PCR analysis of the mRNA expression levels of anti-oxidant genes *Foxo1* (**I**), *Sod1* (**J**), *Sod2* (**K**) and *Cat* (**L**). The relative mRNA expression levels of osteoporotic donor-derived MSCs were obtained by normalizing first against *β-actin* and then against those of the respective healthy donor-derived MSCs. *n* = 6 per group. Data are shown as mean ± SD. *P < 0.05, **P < 0.01 and ***P < 0.001. NS, not significant (*P* > 0.05). Data were analysed using two-tailed Student’s t-test for BMMSCs or ADMSCs, respectively.

The above results collectively revealed a general maintenance of cellular function of ADMSCs, but not BMMSCs, in diseased microenvironments, which prompted us to further dissect the mechanisms regarding the general cell status. It has been documented that stemness loss of MSCs determines their impaired function and therapeutic efficacy in detrimental conditions^33^. Accordingly, we examined the mRNA expression levels of stemness markers: *Nanog*^34^, *Sex determining region Y-box 2* (*Sox2*)^35^, *C-myc*^36^ and *Kruppel like factor 4* (*Klf4*)^37^. The results demonstrated that ADMSCs maintained stemness when derived from diseased microenvironments, while osteoporotic donor-derived BMMSCs showed reduction in stemness (Fig. 6A–D).

Both glycolysis and oxidative phosphorylation (OXHPOS) play an important role in MSCs. Despite that MSCs rely more on glycolysis for energy supply under quiescent condition, oxidative phosphorylation (OXHPOS) becomes critically important for MSCs upon osteogenic differentiation^38^, the impairment of which by inflammation causes function decline of MSCs^2,39^. Furthermore, decline of antioxidant defence caused by accumulation of pro-inflammation cytokines after estrogen deficiency induces oxidative damage in BMMSCs, leading to a defect of bone formation in osteoporosis^40^. Therefore, we focused on both glycolysis and OXHPOS as well as antioxidant defence system to evaluate the cellular statue of MSCs. We have found that BMMSCs and ADMSCs derived from healthy donors displayed comparable glycolysis and OXHPOS status (Fig. 6E,F**)** as well as intracellular ATP and ROS contents (Supplementary Figure S6), As to MSCs derived from osteoporotic donors, dADMSCs possessed similar profiles of glycolysis and OXPHOS to those of hADMSCs and hBMMSCs while dBMMSCs displayed lowered glycolysis and OXPHOS levels (Fig. 6E,F). We further investigated the mRNA expression levels of OXHPOS genes *Respiratory chain complex II* (*CII)* and *Respiratory chain complex V* (*CV*)^41^, and the results showed that energy metabolism was preserved in dADMSCs while inhibited in dBMMSCs (Fig. 6G,H). Meanwhile, as demonstrated by the mRNA expression levels of key anti-oxidant genes *Superoxide dismutase* 1 (*Sod1*) and *Catalase* (*Cat*)^42^, osteoporotic donor-derived ADMSCs, but not BMMSCs, exhibited retained anti-oxidative defence system. Similar tendency was also detected in the expression levels of other anti-oxidant genes *Forkhead box O1* (*Foxo1*)^40^ and *Superoxide dismutase 2* (*Sod2*)^43^, though without statistical significance (Fig. 6I–L). Taken together, these data suggested a general preservation of cellular states regarding the retained stemness, metabolism and anti-oxidants of ADMSCs in diseased microenvironments.

## Discussion

Maintenance of bone homeostasis against diseased microenvironments remains as a major challenge in medical science^2,44^. Recently, systemic MSC therapy has emerged as a promising non-invasive approach for prevention and rescue of extensive bone loss. However, one of the most intricate issues that restrict further application of MSC therapy is the reciprocal impacts of diseased microenvironments on function and therapeutic potential of the current popular cell candidate, BMMSCs^3,9^. In this study, we uncovered discrepancies regarding the responses to common microenvironmental conditions and the preservation of microenvironmental modulatory capacities between BMMSCs and an alternative subpopulation, ADMSCs. We discovered that, unlike BMMSCs, ADMSCs derived from estrogen-deficient diseased donors retained their anti-inflammatory potential to maintain bone homeostasis upon systemic infusion into estrogen-deficient recipients. Furthermore, we revealed that, in contrast to BMMSCs, the general cellular states of ADMSCs, including stemness, energy metabolism and anti-oxidative defence system, were not impaired by diseased microenvironments, enabling preservation of their capability to regulate T-cell viability. This functional resistance of ADMSCs to diseased microenvironments probabilizes them to exert microenvironmental modulatory effects as an optimal source for osteoporotic cytotherapy.

It is well known that osteoporosis develops because of imbalanced bone remodelling, which is attributed to various pathological factors that influence cell behaviours^1^. It is further recognized in estrogen deficiency-induced osteoporosis that osteoclastic number and activity are stimulated while osteoblastic parameters are decreased, as also shown by our data, due to lack of estrogen and the secondary onset of inflammation^2^. In our previous study, we have reported that BMMSC therapy rescues the disordered bone remodelling in OVX mice, restoring both osteoclasts and osteoblasts^9^. Here, we further revealed that osteoporotic donor-derived ADMSCs, but not BMMSCs, preserved their protective effects on bone mass and bone remodelling balance in osteoporosis. Despite the possibility that MSCs may affect bone remodelling through local effects such as differentiation, only a few systemically infused MSCs could home into recipient bone marrow. Furthermore, our previous study have found that most donor MSCs could not be detected during the experimental period, indicating that the recovered osteoblasts are probably not of donor origin^9^. Therefore, the main therapeutic mechanism underlying MSC therapy is proposed to be systemic effects. Specifically, the newly formed bone after MSC therapy should be attributed to systemic microenvironmental modulation that indirectly rescues osteoblastic bone formation.

According to our previous work basis^3,9,24^, the main pathogenic mechanism underlying estrogen deficiency-induced osteoporosis is excessive pro-inflammatory cytokines, such as TNF-α and INF-γ, which is primarily due to the increase of the total T-cell population. Accordingly, we and others have revealed that through anti-inflammation via induction of T-cell apoptosis, BMMSC infusion is enough to treat OVX osteopenia in mice^8,9^. Nevertheless, it has gradually been noticed that host comorbidities of microenvironmental statuses greatly influence the therapeutic performance of MSCs; especially, we have previously revealed that the anti-inflammatory capability of BMMSCs could be easily manipulated by recipient microenvironmental changes in osteoporotic cytotherapy^9^. In the present study, we further demonstrated that BMMSCs are indeed prone to diseased microenvironments and lost their anti-inflammatory capability to prevent bone loss and rescue bone remodelling balance in osteoporosis. On the contrary, ADMSCs preserved their anti-inflammatory capacity, and continued to show protective effects on bone mass and bone remodelling balance in osteoporosis, shedding light on applications in future translational experiments.

Previously, we have revealed that the pivotal driving force for pro-inflammatory cytokines accumulation in estrogen deficiency-induced osteoporosis is the increase of the total T-cell population^8,9^. Accordingly, we have explicated that systemically infused BMMSCs alleviated ovariectomized osteopenia in mice via inducing T-cell apoptosis which further decreased pro-inflammatory cytokines^8,9^. Therefore, although other changes of T cells such as suppressed antigen responses, functional alterations among different T-cell subpopulations and subtype switch of T cells may also contribute to anti-inflammation of MSCs, T-cell viability modulations is the primary mechanism underlying MSC-mediated treatment for osteoporosis^7,8^. The reason underlying differential anti-inflammation performances of diseased donor-derived MSCs in diverse conditions is not understood; nonetheless, in this study, we showed that at least reduction of critical immunomodulatory factors Fasl^7,8^, Ido^27^ and Mmp9^28^ contributed to functional decline of anti-inflammation of osteoporotic BMMSCs. Importantly, we discovered that osteoporotic donor-derived ADMSCs retained expression levels of these immunomodulatory factors, underlying their superiority over osteoporotic donor-derived BMMSCs in anti-inflammatory capacity and efficacy to maintain bone homeostasis in recipient estrogen deficiency.

The adipose tissue represents a promising resource of MSCs with abundant storage and convenience to harvest^45^. Furthermore, ADMSCs have been uncovered proliferation and differentiation properties with immense bone regenerative potential, which are more importantly preserved when derived from osteoporotic donors^12,15–19^. In this study, we further identified that anti-inflammatory capability of ADMSCs was also retained in osteoporotic development and cytotherapy, suggesting functional resistance to diseased microenvironments of bone while the anti-inflammation ability of BMMSCs is impaired by diseased microenvironment. It is interesting that the two MSC subpopulations exhibit functional discrepancies in responses to the common microenvironmental impacts. We have previously documented that under estrogen deficiency, the secondary onset of circulatory immunological disorders leads to uniform elevation of inflammation in local microenvironments of different tissues^3^. Therefore, detailed response patterns of ADMSCs and BMMSCs to the specific microenvironmental signals of inflammatory cytokines should be clarified in future studies. Molecularly, it has been demonstrated that ADMSCs and BMMSCs harness differential transcriptional regulation and posttranscriptional regulation networks^46–48^. Here, we recognized even more fundamental mechanistic discrepancies regarding general cellular states of stemness and metabolism.

Our group has previously revealed that stemness loss of BMMSCs leads to function decline and failure of BMMSCs therapy, while recovery of stemness improves the functionality and treatment efficacy of BMMSCs^33^. In this study, we further elucidated that ADMSCs maintained stemness when derived from diseased microenvironments, while osteoporotic donor-derived BMMSCs showed reduction in stemness, which is supposed to be underlying the discrepancy between ADMSCs and BMMSCs. For metabolism of MSCs, in estrogen deficiency-induced osteoporosis, the decline of OXPHOS leads to suppressed generation of ATP with upregulated oxidative stress in osteoporotic BMMSCs, resulting in impaired function (unpublished data)^40^. Our study revealed that differences of dADMSCs and dBMMSCs were at least partially due to their discrepancies of metabolic status, as shown by glycolysis and OXPHOS levels, which was further confirmed by analysis of mitochondrial complex and antioxidant system integrity. Further elucidating the potential regulatory axis from posttranscriptional regulators to signalling pathways and gene transcription thus determining the general cellular states would provide better understanding regarding the nature of these cells and insights of the origin-specific response patterns of MSCs to extrinsic signals.

In summary, ADMSCs resist detrimental impacts of diseased donor microenvironments, preserving anti-inflammatory capability to modulate recipient microenvironments in maintenance of bone homeostasis via systemic infusion. Collectively, our findings optimize osteoporotic cytotherapy by using ADMSCs in resistance to and in modulation of diseased microenvironments.

## Methods

### Animals

All experiments were performed in accordance with the Guidelines of Intramural Animal Use and Care Committee of Fourth Military Medical University and the ARRIVE guidelines. Furthermore, all the experimental protocols were approved by School of Stomatology, Fourth Military Medical University (Tab No. 2014037).

12-week-old female wild type (WT) C57BL/6 mice (Laboratory Animal Center, Fourth Military Medical University, China) were applied. For OVX model establishment, mice underwent anaesthesia and received either bilateral OVX or a Sham operation at D0. For isolation of Sham and osteoporotic donor-derived MSCs, mice were sacrificed at D28 after operations. For MSC systemic infusion, mice received MSC transplantation at D7 after operations and were modelled for the followed 3 weeks. At D28, mice were sacrificed. The mice were kept with a 12-h light/dark cycle and free access to food and water when alive.

### Isolation, culture, identification and functional tests of MSCs

Isolation and culture of murine BMMSCs and ADMSCs were performed as previously described^48,49^. For BMMSCs, hindlimbs were removed and bone marrow cells were collected by flushing marrow cavities. The adherent cells were cultured in media of alpha-minimum essential medium (α-MEM) supplemented with 10% fetal bovine serum (FBS), 2-mM L-glutamine, 100-U/mL penicillin, and 100-g/mL streptomycin (all from Invitrogen, USA). For ADMSCs, subcutaneous adipose tissues were minced, digested by collagenase type I (Invitrogen, USA) and filtered through cell strainer. After centrifugation, the deposit was resuspended in media same as BMMSCs. Cells were cultured in a humidified atmosphere of 5% CO_2_ at 37 °C. The cells were digested and passaged using 0.25% trypsin (Invitrogen, USA).

MSCs at the 1^st^ passage were used for surface makers identification and colony formation, proliferation and multilineage differentiation assays, as previously described with minor modifications^25^. For surface marker profiling, MSCs of 1 × 10^6^ cells/ml were suspended in PBS supplemented with 3% FBS. 1-μl antibody of CD11b, CD29, CD34, CD45 and Stem cell antigen-1 (Sca-1) (all from Abcam, UK) were respectively added to 2 × 10^5^ cells/tube. After incubation, MSCs were washed twice with PBS and the positively stained cells were detected by a flow cytometer (CytoFLEX; Beckman Coulter, USA) equipped with the CXP 2.1 software.

For colony-forming efficiency (CFE) assay, 1 × 10^4^ MSCs were seeded in a 10-cm dish and cultured for 2 weeks. The colonies were fixed by 4% paraformaldehyde (Sigma-Aldrich, USA), and stained with crystal violet. Colonies with over 50 cells were counted, and raw counts data were presented for analyses.

For cell proliferation analyses, MSCs were seeded at 2 × 10^3^ cells/well in 96-well plates and assessed with a cell counting kit-8 (CCK-8 Kit; Dojindo, Japan)^33,50^. At the same time point from Day-0 to Day-6, cells were incubated with CCK-8 solution for 2 h. Then, the absorbance was measured by a microplate reader (Bio-TEK Instruments; Winooski, USA) at the optical density (OD) of 450 nm. The proliferation was depicted by the OD_450_ curves, in which OD values of each day were normalized to those of Day-0. The statistical analysis of the cell viability was performed at Day-3, with the relative viability of osteoporotic donor-derived MSCs normalized against that of healthy donor-derived MSCs.

For osteogenic and adipogenic differentiation assays, MSCs were plated at 2 × 10^5^ cells/well in 12-well plates and incubated with osteogenesis-inducing or adipogenesis-inducing media, as stated before^25^. After 7 days of osteogenic induction, ALP staining was performed with a commercial kit (Beyotime, China). Photos were taken and the grey values were quantified with ImageJ 1.47 software. After 14 days of induction, mineralized nodules were stained with alizarin red (Sigma-Aldrich, USA) which were dissolved with cetylpyridinium chloride (Sigma-Aldrich, USA), and quantified by a microplate reader (Bio-TEK Instruments; Winooski, USA) at 570 nm^51^. After 7 days of adipogenic induction, lipid droplet formation was determined by oil red O (Sigma-Aldrich, USA) staining which were dissolved with 60% isopropanol (Sigma-Aldrich, USA), and quantified by a microplate reader (Bio-TEK Instruments; Winooski, USA) at 520 nm^51^. The relative ALP activity, mineralization and lipid droplet formation of osteoporotic donor-derived MSCs was obtained by normalizing against those of healthy donor-derived MSCs.

### Metabolic analysis

Glycolysis rate was determined via assessment of extracellular acidification with an Extracellular pH Sensor Kit based on the manufacturer’s instructions (ENZO Life Sciences, USA). Briefly, 1^st^-passaged MSCs were added with Extracellular pH Sensor Probe and measured using a fluorescence plate reader (Fluoroskan Ascent; Thermo Scientific, USA) for 120 minutes, with fluorescence values of each timepoint normalized to those of 0 min.

OXPHOS rate was determined via assessment of extracellular O_2_ with an O_2_ Extracellular Sensor Kit based on the manufacturer’s instructions (ENZO Life Sciences, USA)^52^. Briefly, 1^st^-passaged MSCs were added with Extracellular O_2_ Sensor Probe and measured using a fluorescence plate reader (Fluoroskan Ascent; Thermo Scientific, USA) for 120 minutes, with fluorescence values of each timepoint normalized to those of 0 min.

ATP was measured using an ATP Assay Kit, according to the manufacturer’s instructions (Beyotime, Shanghai, China). Briefly, 1^st^-passaged MSCs were plated in 12-well plates and homogenized in an ice-cold ATP-releasing buffer 24 h later. After centrifugation, the supernatant was added to ATP detection buffer for measurement via a luminometer (GloMax® 20/20 Luminometer; Promega, USA). ATP content was quantified according to the standard curve and normalized by protein concentration.

Intracellular ROS was measured using an ROS Assay Kit (Genmed Scientifics Inc, USA). For fluorescence analysis, 1^st^-passaged MSCs were plated in 12-well plates, incubated with staining reagent and examined under a fluorescence microscope (DP70; Olympus, Japan). For flow cytometric analysis, digested MSCs were incubated with staining reagent and analysed using a flow cytometer (CytoFLEX; Beckman Coulter, USA) equipped with the CXP 2.1 software. The intracellular ROS of ADMSCs was obtained by normalizing against that of BMMSCs.

### T-cell culture and co-culture assays with MSCs

According to previous studies^7,9^, murine spleen cells were collected and removed of red blood cells by ACK lysis buffer (Lonza, Switzerland). Then, T cells were stimulated with 3-μg/mL plate-bound anti-mouse CD3 antibody (eBioscience, USA) for 6 h, followed by 2-μg/mL soluble anti-mouse CD28 antibody (eBioscience, USA) for 2 d in the cell culture media in a humidified atmosphere of 5% CO_2_ at 37 °C. For direct co-culture assay, 1^st^-passaged MSCs were seeded at 2 × 10^5^ cells/well in 12-well plates for 24 h. Stimulated T cells were either cultured without MSCs or directly added at 2 × 10^6^ cells/well onto MSCs for 6 h.

### Systemic infusion of MSCs

MSCs were infused intravenously according to previous reports^8,9^. BMMSCs and ADMSCs from both Sham and OVX donors were transplanted, respectively denoted as hBMMSCs and hADMSCs (from healthy donor microenvironment), and dBMMSCs and dADMSCs (from diseased donor microenvironment). The recipient mice were divided into 6 groups (*n* = 6/each): Sham + PBS, OVX + PBS, OVX + hBMMSCs, OVX + hADMSCs, OVX + dBMMSCs and OVX + dADMSCs. According to previous studies, we chose 1 × 10^6^ BMMSCs per mice as the injection dose, which was a safe dose for mice with high efficacy in osteoporosis treatment^9,53^. Specifically, MSCs at 5 × 10^6^/mL were suspended in PBS and each recipient mouse received 200-μL PBS containing 1 × 10^6^ cells or equivalent PBS via caudal vein. 21 days after infusion, mice were sacrificed for the below analyses of bone and serum examinations.

### Micro-CT analysis

Evaluation of trabecular and cortical bone was performed using a desktop micro-CT system (eXplore Locus SP; GE Healthcare, USA)^25,54^. Upon sacrifice at D28 post operation, the right femora were removed and fixed overnight in 4% paraformaldehyde (Sigma-Aldrich, USA). Bone specimens were then prepared as 1-mm blocks with the distal femora included and scanned at 8-μm resolution, 80-kV voltage and 80-μA current. Trabecular region of interest was defined as a cylindrical region in the distal metaphysis, from 0.3 mm to 0.6 mm away from the epiphysis. Cortical region of interest was defined in the midshaft with the trabecular region subtracted, from 3.3 mm to 3.6 mm away from the epiphysis. Parameters of bone volume per tissue volume (BV/TV), bone mineral density (BMD), trabecular thickness (Tb.Th), trabecular number (Tb.N), trabecular separation (Tb.Sp), cortical thickness (Ct.Th) and cortical area per total area (Ct.Ar/% Tt.Ar) were quantified and analysed with the Micview V2.1.2 software, as recommended^55^.

### Bone histomorphometric analyses

Calcein labelling was applied to examine bone formation rates, as stated before^9,25^. Mice received intraperitoneal injections of calcein (20 mg/kg, Sigma-Aldrich, USA) at 16 d and 2 d prior to sacrifice. At sacrifice, the right femora were isolated, fixed in 80% ethanol, and embedded in methyl methacrylate. The specimens were sagittally cut into 30-μm thick sections and both double-labelled and single-labelled cortical endosteum was detected using a fluorescence microscope (STP6000; Leica, Germany) with an excitation wavelength of 488 nm. Quantification was determined using the ImageJ 1.47 software to evaluate mineral apposition rate (MAR) and bone formation rate (BFR)^9,25^.

TRAP staining was applied to examine bone resorption rates, according to previous studies^9,25^. After being isolated, tibiae were fixed with paraformaldehyde, decalcified with 10% ethylene diamine tetraacetic acid (pH, 7.2-7.4), and embedded in paraffin. The proximal metaphyses were sagittally sectioned into 5-μm sections using a microtome (RM2125; Leica, Germany). The sections were stained with TRAP by a commercial kit according to the manufactures’ instructions (387-1 A; Sigma-Aldrich, USA). Parameters of number of osteoclasts per bone surface (N.Oc/BS) and osteoclast surface per bone surface (Oc.S/BS) were quantified using the ImageJ 1.47 software^9,25^.

### Enzyme-linked immunosorbent assay (ELISA)

For serum examination, at sacrifice, peripheral whole blood was collected from retro-orbital venous plexus and were then centrifuged at 3000 rpm for 10 min followed by 12000 rpm for 10 min at 4 °C. For media examination, conditional media were obtained after T-cell co-culture assays. Murine ELISA kits were used according to the manufacturers’ instructions (R&D Systems, USA). Specifically, bone formation (P1NP and OCN) and bone resorption markers (TRAP-5b and CTX-1) in serum, as well as inflammatory cytokines (TNF-α and IFN-γ) in both serum and media, were detected^8,9,25,54^.

### Detection of T cells in circulation

Peripheral whole blood was collected as stated above. After removal of red blood cells using ACK lysis buffer (Lonza, Switzerland), 1 × 10^6^ cells were incubated with 1-μL PE-labelled anti-mouse CD3 antibody (Biolegend, USA). After washing with PBS twice, flow cytometric analyses of CD3^+^ T cells percentages in peripheral blood mononuclear cells (PBMNCs) were performed using a flow cytometer (CytoFLEX; Beckman Coulter) equipped with the CXP 2.1 software^7,9^.

### Apoptosis analysis

Apoptosis analysis of T cells was performed using Annexin V Apoptosis Detection Kit I (BD Biosciences, USA). Briefly, T cells were harvested after co-culture assays and stained in sequence by FITC-conjugated Annexin V and PI, followed by detection with a flow cytometer (CytoFLEX; Beckman Coulter, USA) equipped with the CXP 2.1 software. The apoptotic rate of T cells was calculated by percentages of early apoptotic (FITC^+^PI^−^) plus late apoptotic/necrotic (FITC^+^PI^+^) cells, as previously documented^9^.

### MSC tracing

To detect the homing of administrated MSCs *in vivo*, ADMSCs and BMMSCs were labelled with PKH67 (Sigma-Aldrich, USA) before injection, according to the producer’s instruction. At 24 h and 3 d after MSCs systemic infusion, femora were collected, fixed in 4% paraformaldehyde and decalcified. Decalcified bones were immersed into 30% sucrose solution, embedded in the optimal cutting temperature compound and sagittally cut into 30-μm sagittal sections^9,25^. After being counterstained with Hoechst, sections were analysed by a fluorescence microscopy (DP70; Olympus, Japan). Images were taken and further analysed by the ImageJ 1.47 software.

### qRT-PCR analysis

Total RNA was collected using Trizol Reagent (Takara, Japan) and purified by phenol-chloroform extraction. 500-ng total RNA was reverse transcribed to cDNA using a PrimeScript RT reagent kit (Takara, Japan), followed by qRT-PCR procedures^2,32^. *β-actin* was used as the internal control. The relative expression levels of genes in osteoporotic donor-derived MSCs were obtained by normalizing against those of healthy donor-derived MSCs. The primer sequences were listed in Supplementary Table S1.

### Statistical analysis

All the data were displayed as the mean ± standard deviation (SD). Comparisons were performed by two-tailed Student’s t-test (for two-group analysis) or one-way analysis of variance (ANOVA) followed by the Newman-Keuls post-hoc tests (for multiple group analysis) using the GraphPad Prism 5.01 software. Significance was confirmed at *P* < 0.05.

## Electronic supplementary material

Supplementary information

## References

[1] Raisz LG (2005). Pathogenesis of osteoporosis: concepts, conflicts, and prospects. J. Clin. Invest..

[2] Sui BD, Hu CH, Zheng CX, Jin Y (2016). Microenvironmental Views on Mesenchymal Stem Cell Differentiation in Aging. J. Dent. Res..

[3] Wang L (2013). IFN-gamma and TNF-alpha synergistically induce mesenchymal stem cell impairment and tumorigenesis via NFkappaB signaling. Stem Cells..

[4] Lee JH (2016). A systematic review of diagnostic accuracy of vertebral fracture assessment (VFA) in postmenopausal women and elderly men. Osteoporos. Int..

[5] Sui, B. D. *et al.* Stem cell-based bone regeneration in diseased microenvironments: Challenges and solutions. *Biomaterials*, 10.1016/j.biomaterials.2017.10.046 (2017). 10.1016/j.biomaterials.2017.10.046 29122279

[6] Ren G (2008). Mesenchymal stem cell-mediated immunosuppression occurs via concerted action of chemokines and nitric oxide. Cell Stem Cell..

[7] Akiyama K (2012). Mesenchymal-stem-cell-induced immunoregulation involves FAS-ligand-/FAS-mediated T cell apoptosis. Cell Stem Cell..

[8] Liu Y (2014). Transplantation of SHED prevents bone loss in the early phase of ovariectomy-induced osteoporosis. J. Dent. Res..

[9] Sui BD (2017). Recipient Glycemic Micro-environments Govern Therapeutic Effects of Mesenchymal Stem Cell Infusion on Osteopenia. Theranostics..

[10] Arthur A, Rychkov G, Shi S, Koblar SA, Gronthos S (2008). Adult human dental pulp stem cells differentiate toward functionally active neurons under appropriate environmental cues. Stem Cells..

[11] Sacchetti B (2007). Self-renewing osteoprogenitors in bone marrow sinusoids can organize a hematopoietic microenvironment. Cell..

[12] Beane OS, Fonseca VC, Cooper LL, Koren G, Darling EM (2014). Impact of aging on the regenerative properties of bone marrow-, muscle-, and adipose-derived mesenchymal stem/stromal cells. Plos One..

[13] Sundh D (2016). A High Amount of Local Adipose Tissue Is Associated With High Cortical Porosity and Low Bone Material Strength in Older Women. J. Bone. Miner. Res..

[14] Gomori A (2007). Blockade of MCH1 receptor signalling ameliorates obesity and related hepatic steatosis in ovariectomized mice. Br. J. Pharmacol..

[15] Veronesi F, Pagani S, Della Bella E, Giavaresi G, Fini M (2014). Estrogen deficiency does not decrease the *in vitro* osteogenic potential of rat adipose-derived mesenchymal stem cells. Age..

[16] Boeloni JN, Ocarino NM, Goes AM, Serakides R (2014). Comparative study of osteogenic differentiation potential of mesenchymal stem cells derived from bone marrow and adipose tissue of osteoporotic female rats. Connect. Tissue. Res..

[17] Zhu M (2009). The effect of age on osteogenic, adipogenic and proliferative potential of female adipose-derived stem cells. J. Tissue. Eng. Regen. Med..

[18] Mirsaidi A (2012). Telomere length, telomerase activity and osteogenic differentiation are maintained in adipose-derived stromal cells from senile osteoporotic SAMP6 mice. J. Tissue. Eng. Regen. Med..

[19] Chen HT (2012). Proliferation and differentiation potential of human adipose-derived mesenchymal stem cells isolated from elderly patients with osteoporotic fractures. J. Cell. Mol. Med..

[20] Stenderup K, Justesen J, Clausen C, Kassem M (2003). Aging is associated with decreased maximal life span and accelerated senescence of bone marrow stromal cells. Bone..

[21] Mirsaidi A (2014). Therapeutic potential of adipose-derived stromal cells in age-related osteoporosis. Biomaterials..

[22] Pei M (2015). A comparison of tissue engineering based repair of calvarial defects using adipose stem cells from normal and osteoporotic rats. Bone..

[23] Ye X (2014). Adipose-derived stem cells alleviate osteoporosis by enhancing osteogenesis and inhibiting adipogenesis in a rabbit model. Cytotherapy..

[24] Yang N (2013). Tumor necrosis factor alpha suppresses the mesenchymal stem cell osteogenesis promoter miR-21 in estrogen deficiency-induced osteoporosis. J. Bone. Miner. Res..

[25] Sui B (2016). Allogeneic Mesenchymal Stem Cell Therapy Promotes Osteoblastogenesis and Prevents Glucocorticoid-Induced Osteoporosis. Stem. Cells. Transl. Med..

[26] Liu Y (2011). Mesenchymal stem cell-based tissue regeneration is governed by recipient T lymphocytes via IFN-gamma and TNF-alpha. Nat. Med..

[27] Meisel R (2004). Human bone marrow stromal cells inhibit allogeneic T-cell responses by indoleamine 2,3-dioxygenase-mediated tryptophan degradation. Blood..

[28] Ding Y (2009). Mesenchymal stem cells prevent the rejection of fully allogenic islet grafts by the immunosuppressive activity of matrix metalloproteinase-2 and -9. Diabetes..

[29] Di Nicola M (2002). Human bone marrow stromal cells suppress T-lymphocyte proliferation induced by cellular or nonspecific mitogenic stimuli. Blood..

[30] Min CK, Kim BG, Park G, Cho B, Oh IH (2007). IL-10-transduced bone marrow mesenchymal stem cells can attenuate the severity of acute graft-versus-host disease after experimental allogeneic stem cell transplantation. Bone. Marrow. Transplant..

[31] Wu T (2015). miR-21 Modulates the Immunoregulatory Function of Bone Marrow Mesenchymal Stem Cells Through the PTEN/Akt/TGF-beta1 Pathway. Stem Cells..

[32] Zhao P (2017). Anti-aging pharmacology in cutaneous wound healing: effects of metformin, resveratrol, and rapamycin by local application. Aging Cell..

[33] Shuai Y (2016). Melatonin Treatment Improves Mesenchymal Stem Cells Therapy by Preserving Stemness during Long-term *In Vitro* Expansion. Theranostics..

[34] Tsai CC, Su PF, Huang YF, Yew TL, Hung SC (2012). Oct4 and Nanog directly regulate Dnmt1 to maintain self-renewal and undifferentiated state in mesenchymal stem cells. Mol. Cell..

[35] Han SM (2014). Enhanced proliferation and differentiation of Oct4- and Sox2-overexpressing human adipose tissue mesenchymal stem cells. Exp. Mol. Med..

[36] Buccini S, Haider KH, Ahmed RP, Jiang S, Ashraf M (2012). Cardiac progenitors derived from reprogrammed mesenchymal stem cells contribute to angiomyogenic repair of the infarcted heart. Basic. Res. Cardiol..

[37] Rustad KC (2012). Enhancement of mesenchymal stem cell angiogenic capacity and stemness by a biomimetic hydrogel scaffold. Biomaterials..

[38] Chen CT, Shih YR, Kuo TK, Lee OK, Wei YH (2008). Coordinated changes of mitochondrial biogenesis and antioxidant enzymes during osteogenic differentiation of human mesenchymal stem cells. Stem Cells..

[39] Sui B, Hu C, Jin Y (2016). Mitochondrial metabolic failure in telomere attrition-provoked aging of bone marrow mesenchymal stem cells. Biogerontology..

[40] Liao L (2016). TNF-alpha Inhibits FoxO1 by Upregulating miR-705 to Aggravate Oxidative Damage in Bone Marrow-Derived Mesenchymal Stem Cells during Osteoporosis. Stem Cells..

[41] Baines HL, Turnbull DM, Greaves LC (2014). Human stem cell aging: do mitochondrial DNA mutations have a causal role?. Aging Cell..

[42] Ebert R (2006). Selenium supplementation restores the antioxidative capacity and prevents cell damage in bone marrow stromal cells *in vitro*. Stem Cells..

[43] Kim H, Lee YD, Kim HJ, Lee ZH, Kim HH (2017). SOD2 and Sirt3 control osteoclastogenesis by regulating mitochondrial ROS. J. Bone. Miner. Res..

[44] Ito H (2014). Clinical considerations of regenerative medicine in osteoporosis. Curr. Osteoporos. Rep..

[45] Schaffler A, Buchler C (2007). Concise review: adipose tissue-derived stromal cells–basic and clinical implications for novel cell-based therapies. Stem Cells..

[46] Bionaz M, Monaco E, Wheeler MB (2015). Transcription Adaptation during *In Vitro* Adipogenesis and Osteogenesis of Porcine Mesenchymal Stem Cells: Dynamics of Pathways, Biological Processes, Up-Stream Regulators, and Gene Networks. PLoS One..

[47] Stockl S, Gottl C, Grifka J, Grassel S (2013). Sox9 Modulates proliferation and expression of osteogenic markers of adipose-derived stem cells (ASC). Cell. Physiol. Biochem..

[48] Su X (2015). MiR-26a functions oppositely in osteogenic differentiation of BMSCs and ADSCs depending on distinct activation and roles of Wnt and BMP signaling pathway. Cell. Death. Dis..

[49] Sui B (2016). Mesenchymal progenitors in osteopenias of diverse pathologies: differential characteristics in the common shift from osteoblastogenesis to adipogenesis. Sci. Rep..

[50] Zhou HS (2018). Lipopolysaccharide impairs permeability of pulmonary microvascular endothelial cells via Connexin40. Microvasc. Res..

[51] Liao L (2013). Redundant miR-3077-5p and miR-705 mediate the shift of mesenchymal stem cell lineage commitment to adipocyte in osteoporosis bone marrow. *Cell*. Death. Dis..

[52] Chu DS (2015). Multivalent display of pendant pro-apoptotic peptides increases cytotoxic activity. J. Control. Release..

[53] Liao L (2017). Heparin improves BMSC cell therapy: Anticoagulant treatment by heparin improves the safety and therapeutic effect of bone marrow-derived mesenchymal stem cell cytotherapy. Theranostics..

[54] Hu CH (2017). miR-21 deficiency inhibits osteoclast function and prevents bone loss in mice. Sci. Rep..

[55] Bouxsein ML (2010). Guidelines for assessment of bone microstructure in rodents using micro-computed tomography. J. Bone. Miner. Res..

