# Supplementary material for: Adipose mesenchymal stem cells from osteoporotic donors preserve functionality and modulate systemic inflammatory microenvironment in osteoporotic cytotherapy
Source: Sci Rep. 2018 Mar 26;8:5215. doi: 10.1038/s41598-018-23098-8 (PMC5980002; doi:10.1038/s41598-018-23098-8)
Supplement: Supplementary file 1 — Supplementary information [file 41598_2018_23098_MOESM1_ESM.pdf]

Supplementary information

Adipose mesenchymal stem cells from osteoporotic donors preserve functionality and modulate systemic inflammatory microenvironment in osteoporotic cytotherapy.

Running Title: ADMSCs resist & modulate diseased microenvironment

Chen-Xi Zheng [1, 2, †], Bing-Dong Sui [1, 2, †], Nu Liu [1, 2, †], Cheng-Hu Hu [1, 3], Tao He [1, 2], Xin-Yi Zhang [1, 2], Pan Zhao [1, 3], Ji Chen [1, 2], Kun Xuan [1, *] and Yan Jin [1, 2, *]

[1] State Key Laboratory of Military Stomatology & National Clinical Research Center for Oral Diseases & Shaanxi International Joint Research Center for Oral Diseases, Center for Tissue Engineering, School of Stomatology, Fourth Military Medical University, Xi'an, Shaanxi, 710032, China

[2] Research and Development Center for Tissue Engineering, Fourth Military Medical University, Xi’an, Shaanxi 710032, China.

[3] Xi’an Institute of Tissue Engineering and Regenerative Medicine, Xi’an, Shaanxi 710032, China.

[†] *Chen-Xi Zheng, Bing-Dong Sui and Nu Liu contributed equally to this work.*

[*] *Corresponding authors: Prof. Yan Jin and Prof. Kun Xuan*

Journal: Scientific Reports

Supplementary information: 6 supplementary figures and 1 supplementary table are provided.

Supplementary Figures and Figure Legends

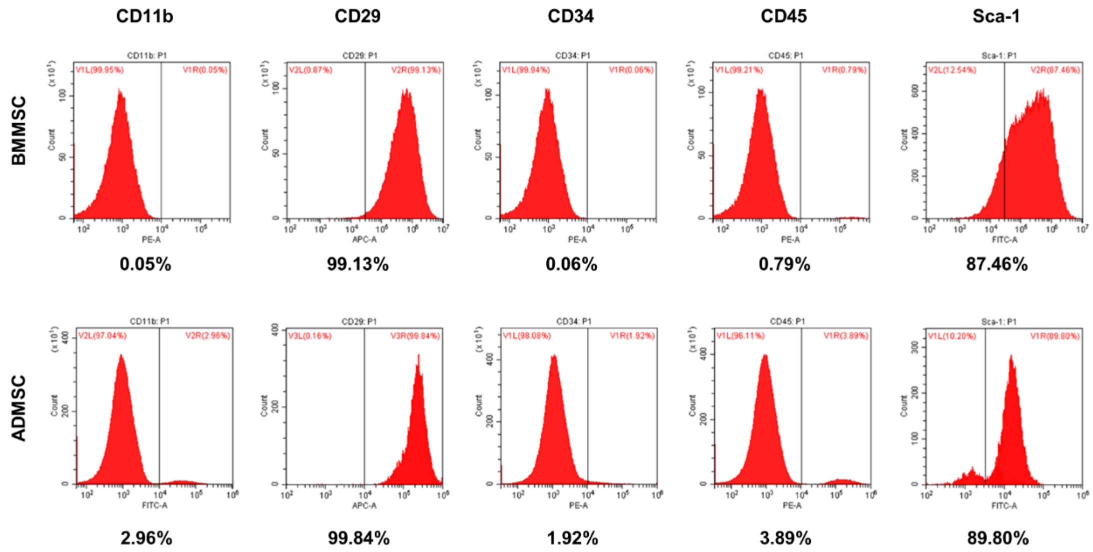

Supplementary Figure S1. Surface marker profiling of MSCs. Flow cytometry analysis of the expression of surface markers: CD11b, CD29, CD34, CD45 and Sca-1 in BMMSCs and ADMSCs.

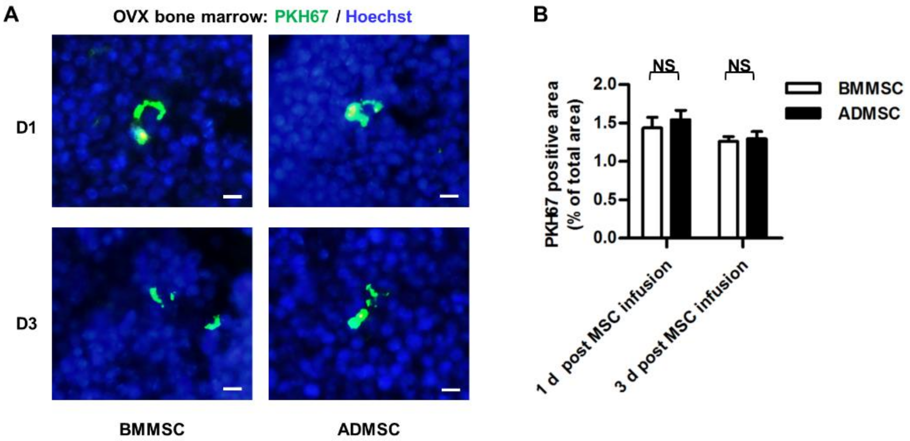

Supplementary Figure S2. Bone marrow tracing of MSCs after systemic transplantation into OVX mice. (**A, B**) Representative images of PKH67 (green) and Hoechst (blue) staining (**A**) with quantification (**B**) of PKH67$^+$ area percentages in bone marrow at D1 and D3 after OVX mice received infusion of PKH67-marked BMMSCs and ADMSCs. Bars: 20 µm. n = 3 per group. Data are shown as mean ± SD. NS, not significant ($P > 0.05$). Data were analysed using two-tailed Student's t-test for D1 or D3, respectively.

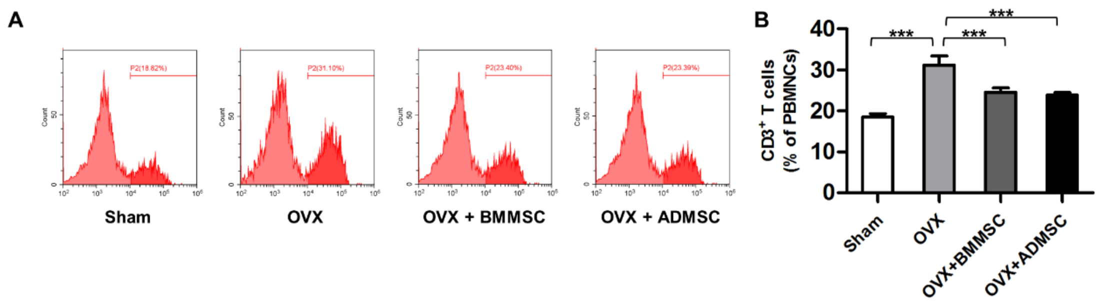

Supplementary Figure S3. Effects of MSCs on $CD3^+$ T cells in peripheral blood of OVX mice. (**A, B**) Representative flow-cytometric images (**A**) and quantitative analysis (**B**) of $CD3^+$ T cell percentages over PBMNCs in Sham and OVX mice receiving PBS or MSCs infusion. $n = 3$ per group. Data are shown as mean ± SD. ***$P < 0.001$. Data were analysed using ANOVA followed by Newman-Keuls post-hoc tests.

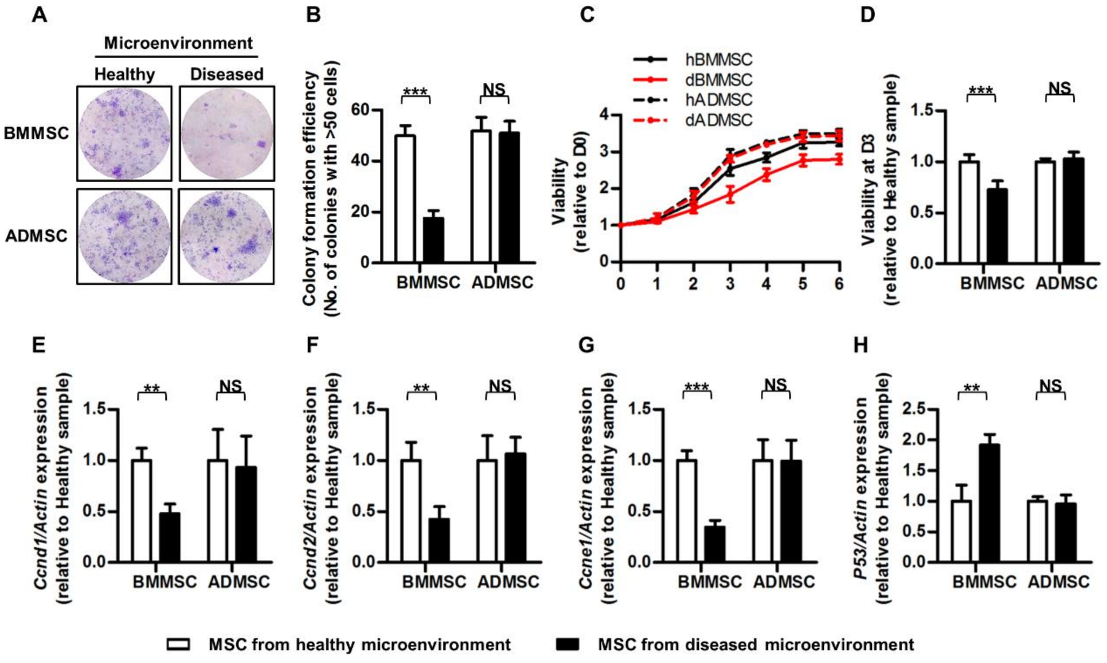

Supplementary Figure S4. Cell viability of MSCs from healthy (Sham donors) and diseased (OVX donors) microenvironments. (**A**) Representative images demonstrating crystal violet-stained colonies formed by ADMSCs and BMMSCs at the 1st passage. (**B**) Quantitation of colony formation efficiency. Colonies with over 50 cells were counted and raw counts were displayed. (**C**) Representative proliferation curves demonstrating proliferation of ADMSCs and BMMSCs at the 1st passage. Cell proliferation was determined with the cell counting kit-8 and depicted by the OD_{450} values. OD values of each day were normalized to those of Day-0. (**D**) Quantitation of cell viability by the OD_{450} values at Day-3. (**E-H**) qRT-PCR analysis of the mRNA expression levels of proliferation-related markers *Ccnd1* (**E**), *Ccnd2* (**F**), *Ccne1* (**G**) and senescence marker *P53* (**H**). The relative parameters of osteoporotic donor-derived MSCs were obtained by normalizing against those of the respective healthy donor-derived MSCs. n = 6 per group. Data are shown as mean ± SD. $^{**}P < 0.01$ and $^{***}P < 0.001$; NS, not significant ($P > 0.05$). Data were analysed using two-tailed Student's t-test for BMMSCs or ADMSCs, respectively.

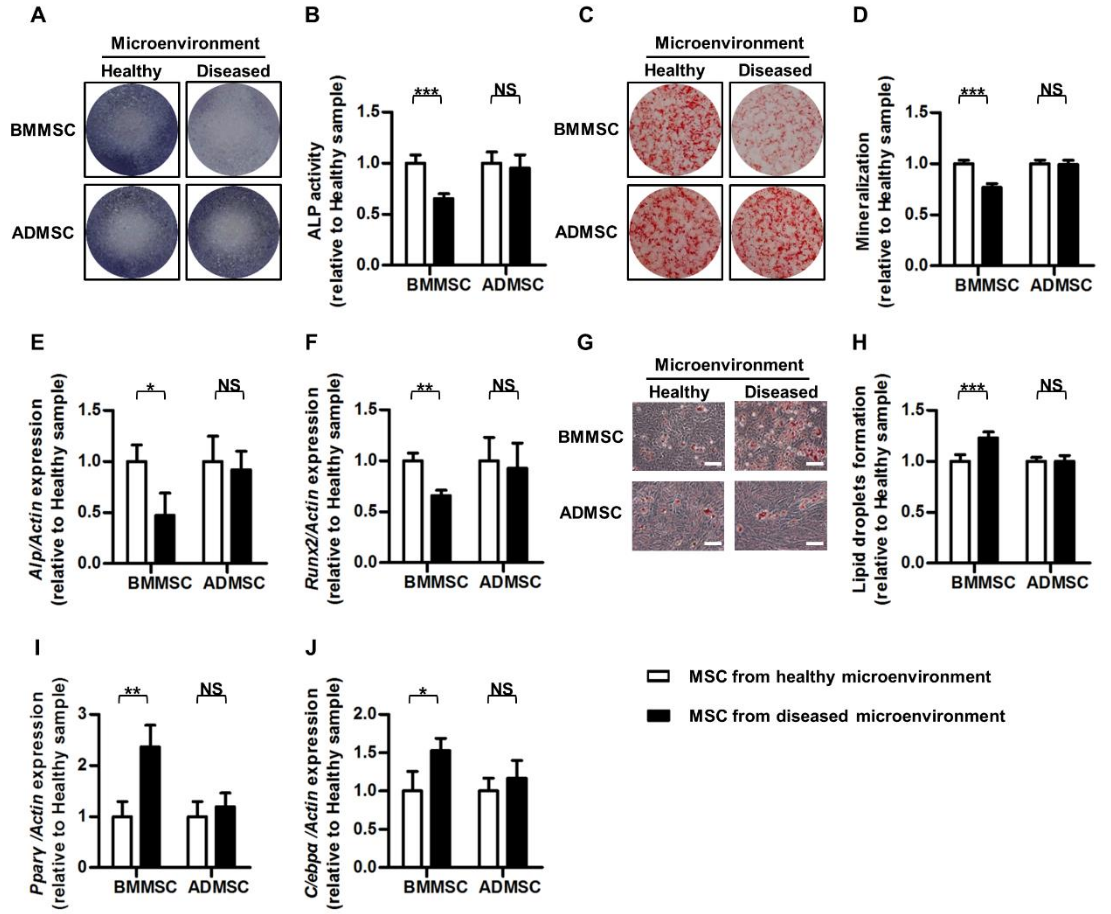

Supplementary Figure S5. Differentiation potential of MSCs from healthy (Sham donors) and diseased (OVX donors) microenvironments. (**A, B**) Representative images of ALP staining and quantitation for grey values. MSCs were induced for osteogenic differentiation at the 1st passage, and ALP activity was examined after induction for 7 days. (**C, D**) Representative images of alizarin red staining and quantitation by the OD_{570} values. MSCs were induced for osteogenic differentiation at the 1st passage, and mineralization was detected after induction for 14 days. The stained mineralized nodules were dissolved with cetylpyridinium chloride and the absorbance was determined. (**E, F**) qRT-PCR analysis of the mRNA expression levels of osteogenic markers *Alp* (**E**) and *Runx2* (**F**). (**G, H**) Representative images of oil red O staining and quantitation by the OD_{520} values. MSCs were induced for adipogenic differentiation at the 1st passage, and lipid droplet formation was detected after induction for 7 days. The stained

lipid droplets were dissolved with 60% isopropanol and the absorbance was determined. Bars: 150 μm. (**I, J**) qRT-PCR analysis of the mRNA expression levels of adipogenic markers *Pparγ* (**I**) and *C/ebpα* (**J**). The relative parameters of osteoporotic donor-derived MSCs were obtained by normalizing against those of the respective healthy donor-derived MSCs. $n = 6$ per group. Data are shown as mean ± SD. *$P < 0.05$, **$P < 0.01$ and ***$P < 0.001$; NS, not significant ($P > 0.05$). Data were analysed using two-tailed Student's t-test for BMMSCs or ADMSCs, respectively.

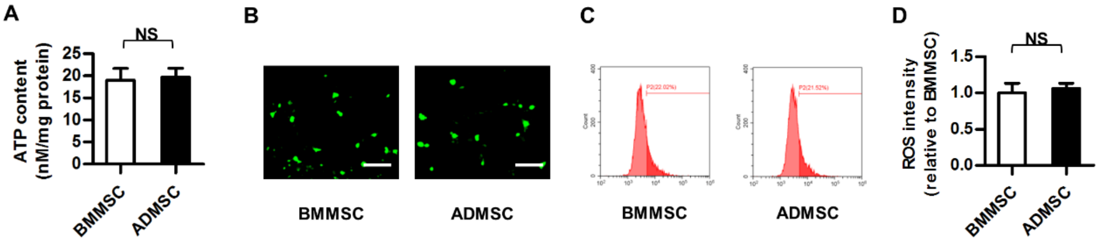

Supplementary Figure S6. Metabolic profiles of BMMSCs and ADMSCs. (**A**) Quantitative analysis of ATP content using the ATP Assay Kit. (**B-D**) Representative images of fluorescence staining (**B**) and flow-cytometric detection (**C**) as well as quantitative analysis (**D**) of ROS contents in MSCs. The relative parameters of ADMSCs were normalized against those of BMMSCs. $n = 3$ per group. Data are shown as mean ± SD. NS, not significant ($P > 0.05$). Data were analysed using two-tailed Student's t-test.

Supplementary Table

Supplementary Table S1. Primer sequences in the present study.

Gene	Primer sequences
β-actin	Forward: 5'-CATCCGTAAAGACCTCTATGCCAAC-3'
	Reverse: 5'-ATGGAGCCACCGATCCACA-3'
Ccnd1	Forward: 5'-AGGCGGATGAGAACAAGCAG-3'
	Reverse: 5'-CCTTGTTTAGCCAGAGGCCG-3'
Ccnd2	Forward: 5'-TACCTCCCGCAGTGTTCCTA-3'
	Reverse: 5'-GCCAAGAAACGGTCCAGGTA-3'
Ccne1	Forward: 5'-AGGCCAAGAAGAGAAAAACAGC-3'
	Reverse: 5'-AGGGCTGATTCCTCCAGACA-3'
P53	Forward: 5'-ATGAACCGCCGACCTATCC-3'
	Reverse: 5'-GGCAGGCACAAACACGAAC-3'
Alp	Forward: 5'-TTGTGCCAGAGAAAGAGA-3'
	Reverse: 5'-GTTTCAGGGCATTTTTCAAGG-3'
Runx2	Forward: 5'-GACTGTGGTTACCGTCATGGC-3'
	Reverse: 5'-ACTTGGTTTTTCATAACAGCGGA-3'
Pparγ	Forward: 5'-ACTGCCGGATCCACAAAA-3'
	Reverse: 5'-TCTCCTTCTCGGCCTGTG-3'
C/ebpα	Forward: 5'-CTGATTCTTGCCAAACTGAG-3'
	Reverse: 5'-GAGGAAGCTAAGACCCACTAC-3'
Nanog	Forward: 5'-CGGTGGCAGAAAAACCAGTG-3'

	Reverse: 5’-AAGGCTTCCAGATGCGTTCA-3’
Sox2	Forward: 5’-ACAGCATGTCCTACTCGCAG-3’
	Reverse: 5’-ATGCTGATCATGTCCCGGAG-3’
C-myc	Forward: 5’-CCTAGTGCTGCATGAGGAGAC-3’
	Reverse: 5’-CTCTTGAGGACCAGTGGGCT-3’
Klf4	Forward: 5’-GCCACCCACACTTGTGACTA-3’
	Reverse: 5’-CTGTGTGTTTGCGGTAGTGC-3’
Sod1	Forward: 5’-GGAAGCATGGCGATGAAAGC-3’
	Reverse: 5’-CCCCATACTGATGGACGTGG-3’
Sod2	Forward: 5’-CAGACCTGCCTTACGACTATGG-3’
	Reverse: 5’-CTCGGTGGCGTTGAGATTGTT-3’
Foxo1	Forward: 5’-AGATGAGTGCCCTGGGCAGC-3’
	Reverse: 5’-GATGGACTCCATGTCACAGT-3’
Cat	Forward: 5’-AGCGACCAGATGAAGCAGTG-3’
	Reverse: 5’-TCCGCTCTCTGTCAAAGTGTG-3’
C Ⅱ	Forward: 5’-GACCCGCTTATGTGTCAGCA-3’
	Reverse: 5’-AGTGGAGAGATGCAGCCTTG-3’
C Ⅴ	Forward: 5’-GCCAGAGACTAGGACTGGAGA-3’
	Reverse: 5’- AGACTGTTCCAATACCAGCAC-3’
Fasl	Forward: 5’-GGCTCTGGTTGGAATGGGAT-3’
	Reverse: 5’-AAATGGGCCACACTCCTCG-3’
Hgf	Forward: 5’-CCACCATAATCCCCCTCACA-3’

	Reverse: 5’-GGCTGGGGCTACACTGGATT-3’
Ido	Forward: 5’-CCAGTGCAGTAGAGCGTCAA-3’
	Reverse: 5’-TCCCAGACCCCCTCATACAG-3’
Il10	Forward: 5’-GCCGGGAAGACAATAACTGC-3’
	Reverse: 5’-AAGGCTTGGCAACCCAAGTA-3’
Inos	Forward: 5’-CAGATCGAGCCCTGGAAGAC-3’
	Reverse: 5’-CAACCTTGGTGTTGAAGGCG-3’
Mmp2	Forward: 5’-CCCCATGAAGCCTTGTTTACC-3’
	Reverse: 5’-GAAGGGGAAGACACATGGGG-3’
Mmp9	Forward: 5’-CCATGCACTGGGCTTAGATCA-3’
	Reverse: 5’-GGCCTTGGGTCAGGCTTAGA-3’
Tgfβ1	Forward: 5’-AGGGCTACCATGCCAACTTC-3’
	Reverse: 5’-CCACGTAGTAGACGATGGGC-3’
